# Supplementary material for: Gallic Acid Alkyl Esters: Trypanocidal and Leishmanicidal Activity, and Target Identification via Modeling Studies
Source: Molecules. 2022 Sep 10;27(18):5876. doi: 10.3390/molecules27185876 (PMC9501172; doi:10.3390/molecules27185876)
Supplement: Supplementary file 1 [file molecules-27-05876-s001.zip › molecules-1892001-supplementary.pdf]

Supplementary Material

Gallic Acid Alkyl Esters: Trypanocidal and Leishmanicidal Activity, and Target Identification via Modeling Studies

Dietmar Steverding [1,*], Lázaro Gomes do Nascimento [2], Yunierkis Perez-Castillo [3,4] and Damião Pergentino de Sousa [2,*]

[1] Bob Champion Research and Education Building, Norwich Medical School, University of East Anglia, Norwich NR4 7UQ, UK
[2] Laboratory of Pharmaceutical Chemistry, Department of Pharmaceutical Sciences, Federal University of Paraíba, João Pessoa 58051-900, PB, Brazil
[3] Bio-Cheminformatics Research Group, Universidad de Las Américas, Quito 170516, Ecuador
[4] Facultad de Ingeniería y Ciencias Aplicadas, Área de Ciencias Aplicadas, Universidad de Las Américas, Quito 170516, Ecuador
* Correspondence: d.steverding@uea.ac.uk (D.S.); damiao_desousa@yahoo.com.br (D.P.d.S.)

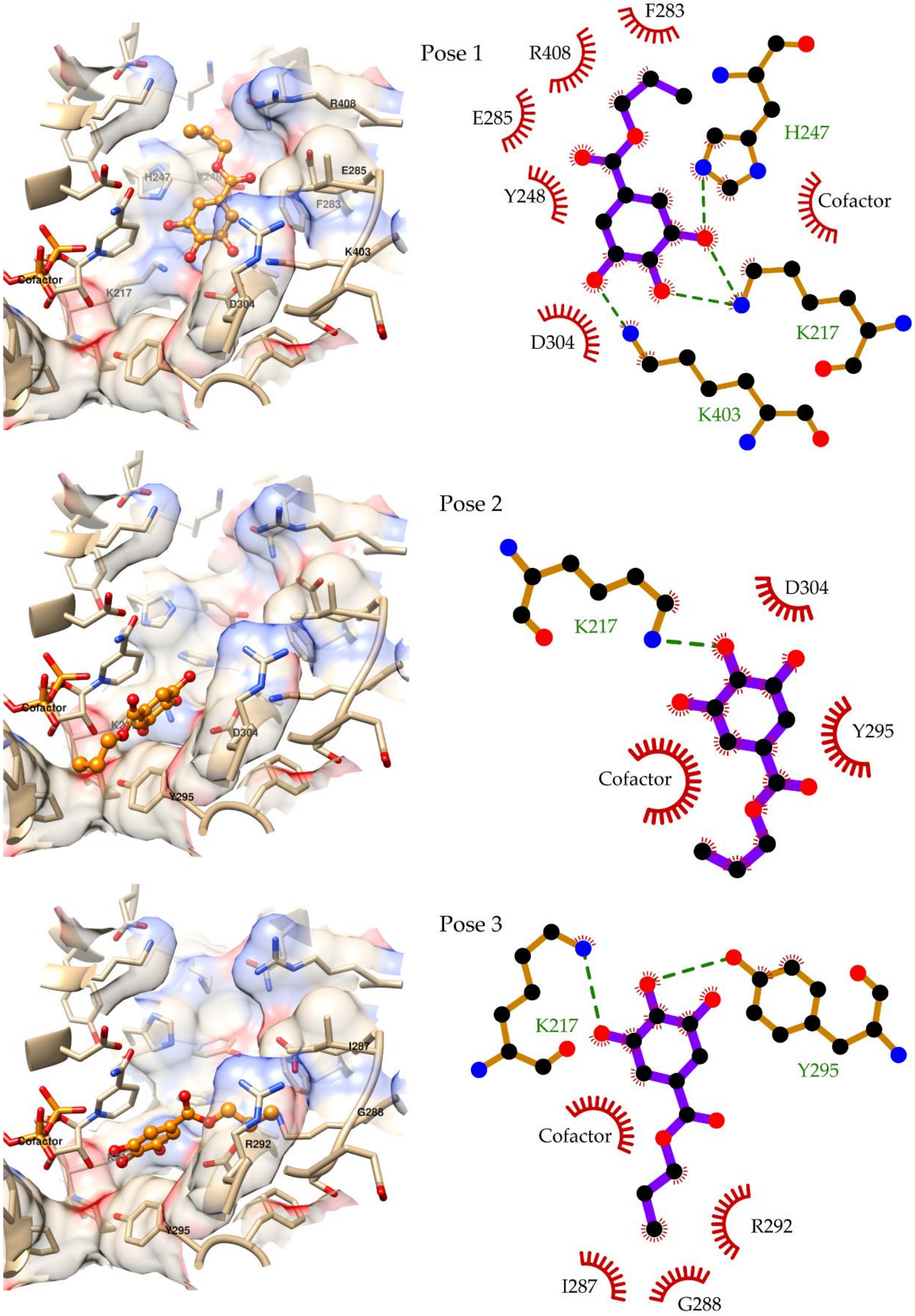

Figure S1. Predicted binding modes and networks of intermolecular interactions obtained from the molecular docking of compound **4**-to the substrate binding site of G6PD. Compound **4** is colored orange and represented as balls and sticks.

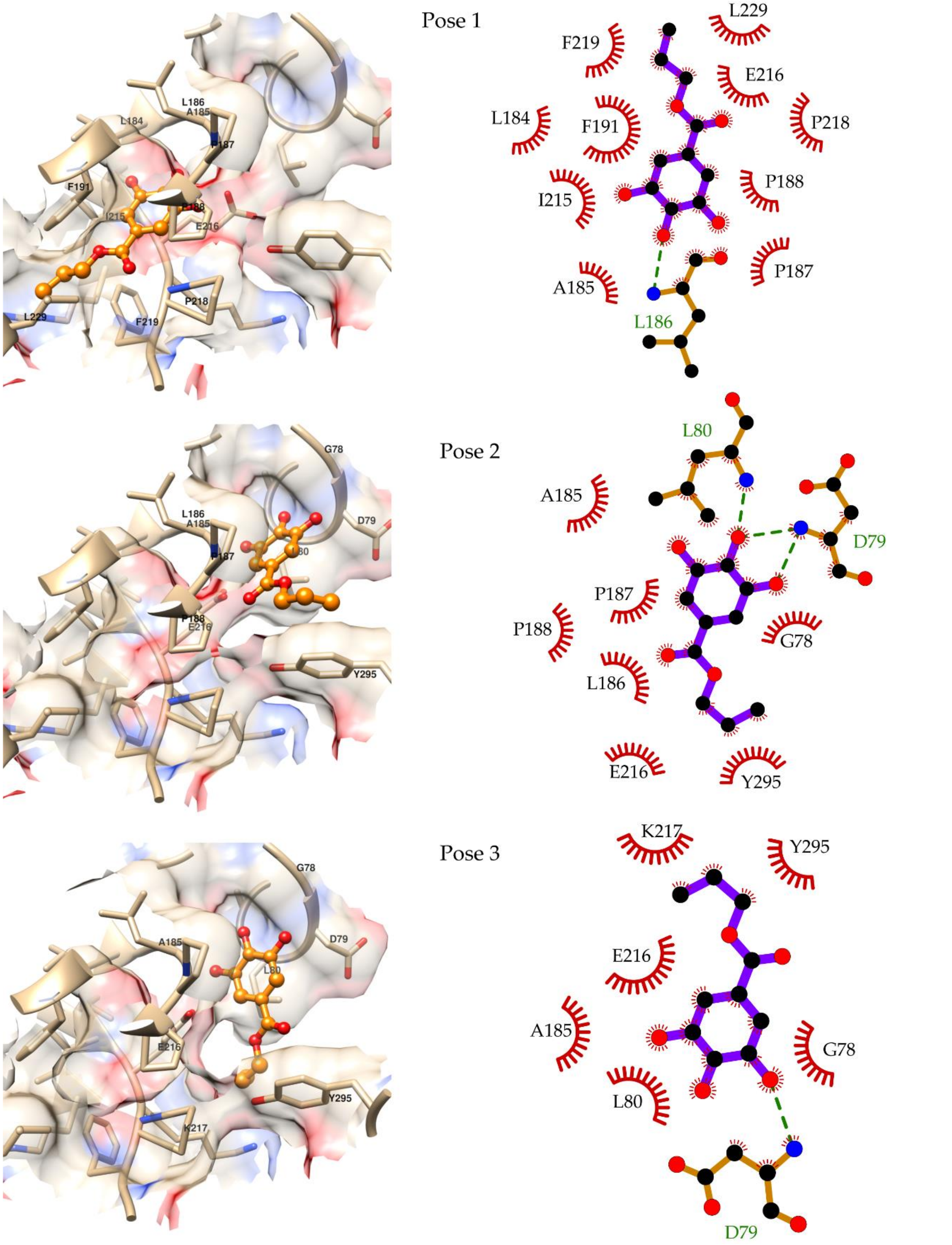

Figure S2. Predicted binding modes and networks of intermolecular interactions obtained from the molecular docking of compound **4**-to the cofactor binding site of G6PD. Compound **4** is colored orange and represented as balls and sticks.

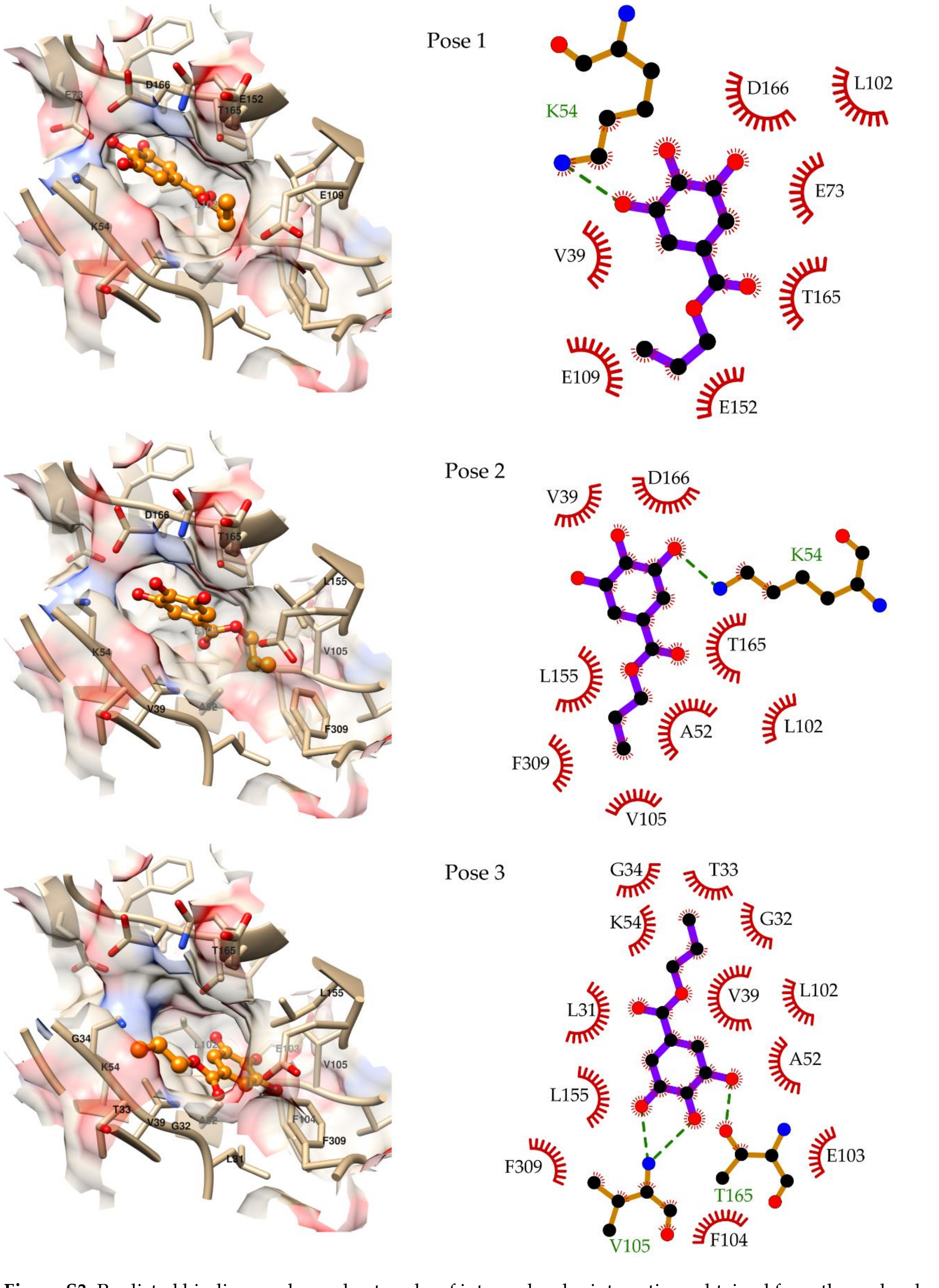

Figure S3. Predicted binding modes and networks of intermolecular interactions obtained from the molecular docking of compound **4**-to PKA1. Compound **4** is colored orange and represented as balls and sticks.

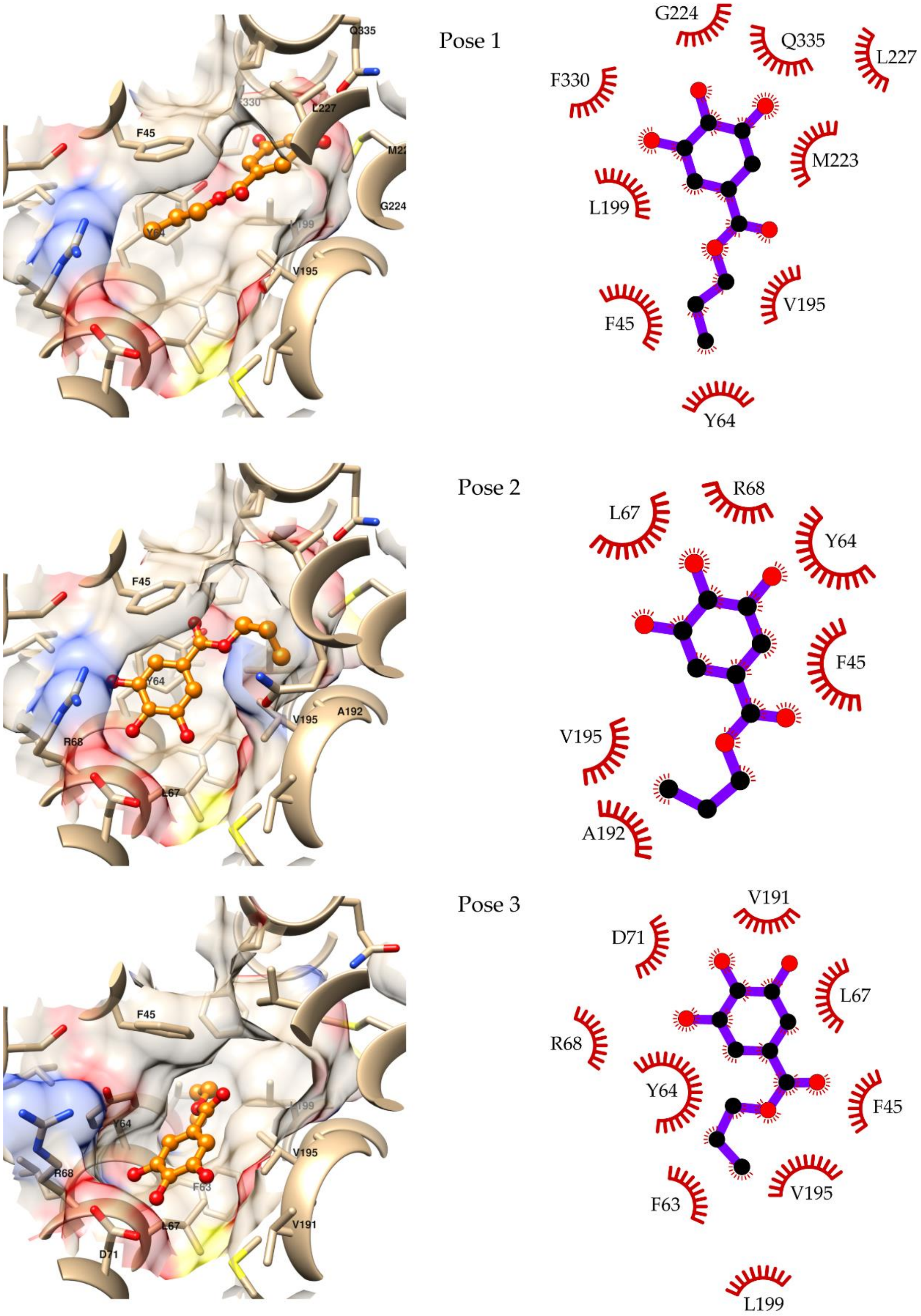

Figure S4. Predicted binding modes and networks of intermolecular interactions obtained from the molecular docking of compound **4**-to FT. Compound **4** is colored orange and represented as balls and sticks.

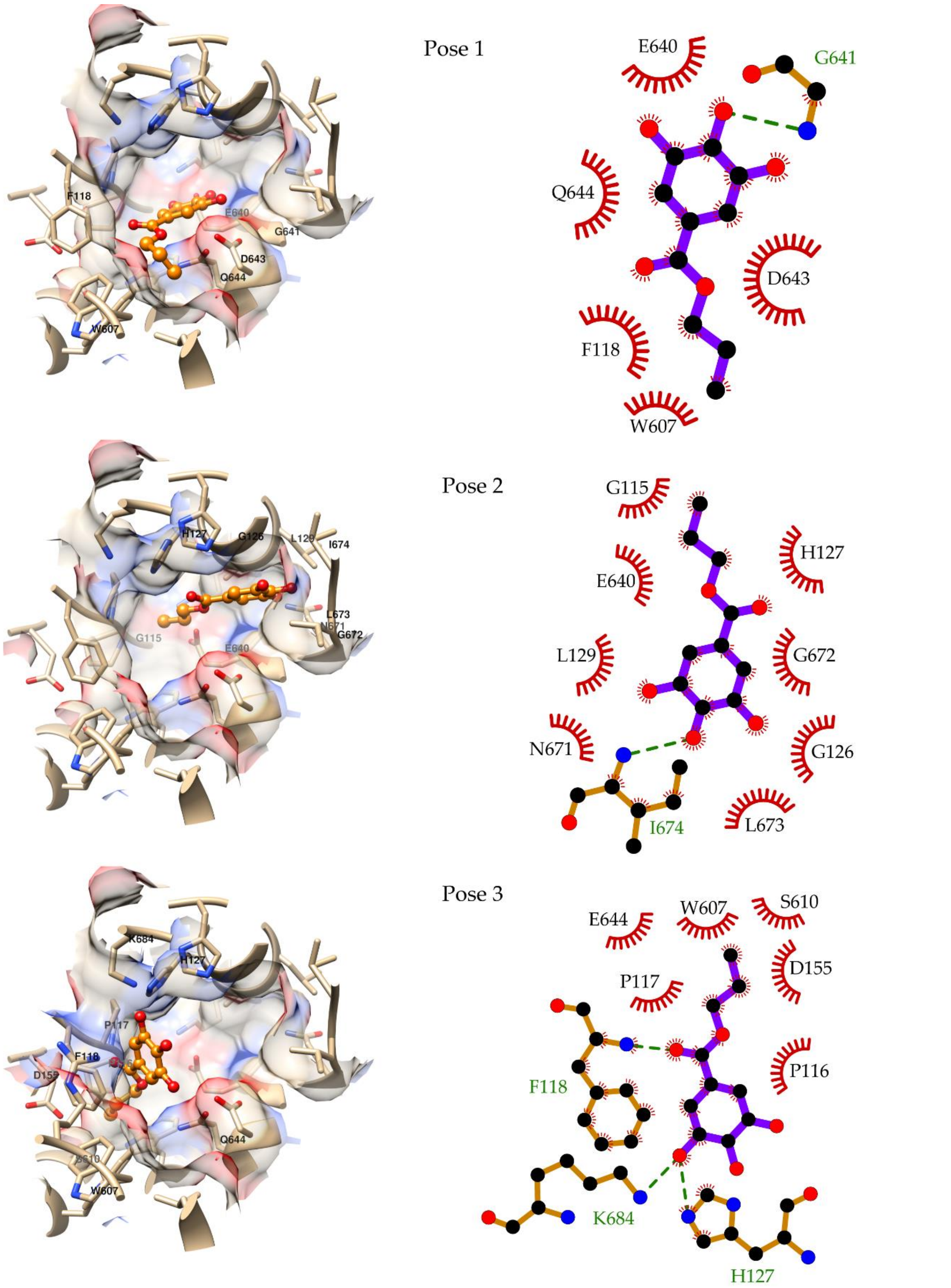

Figure S5. Predicted binding modes and networks of intermolecular interactions obtained from the molecular docking of compound **4**-to IleRL. Compound **4** is colored orange and represented as balls and sticks.

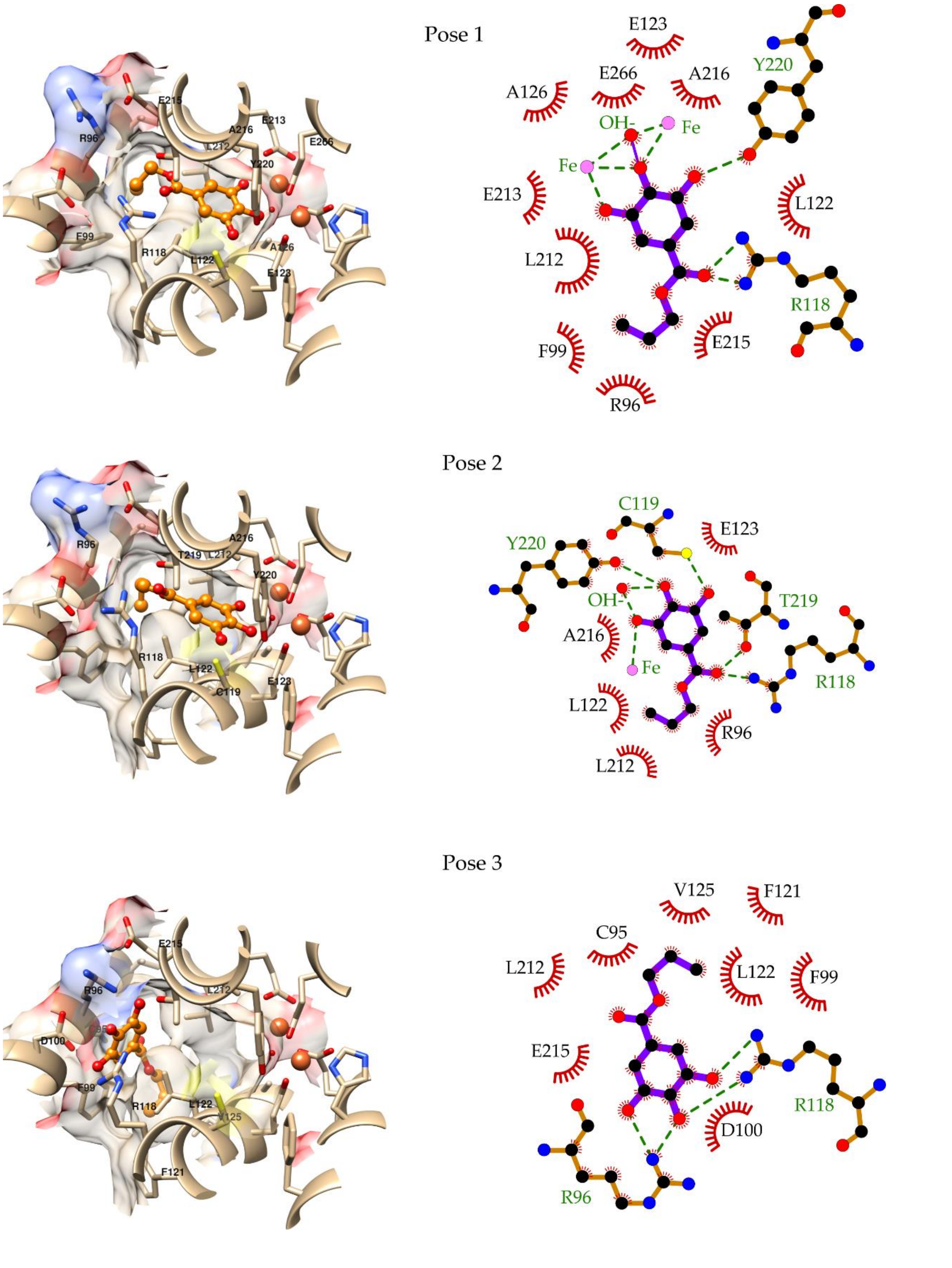

Figure S6. Predicted binding modes and networks of intermolecular interactions obtained from the molecular docking of compound **4**-to TAO in presence of the hydroxide anion. Compound **4** is colored orange and represented as balls and sticks.

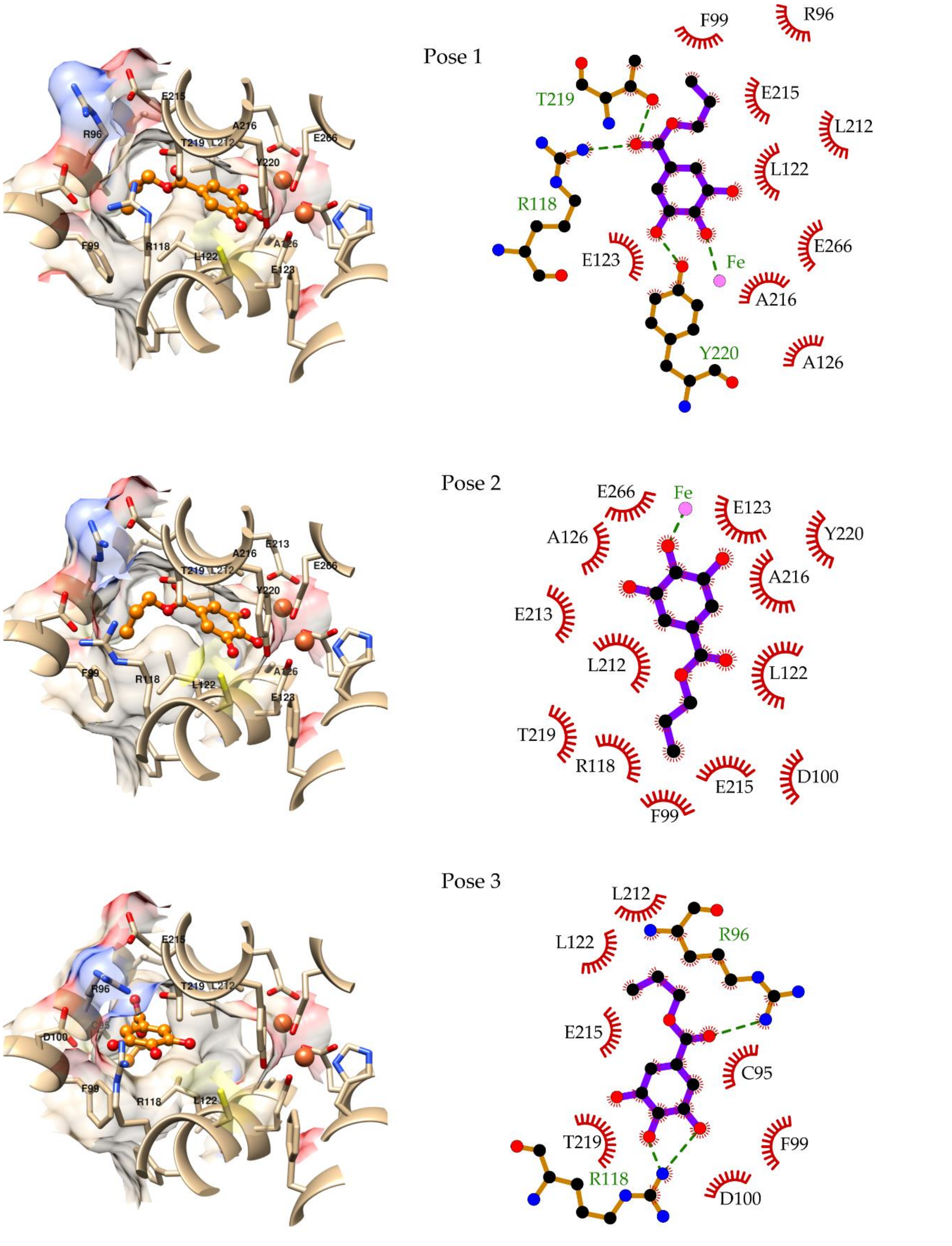

Figure S7. Predicted binding modes and networks of intermolecular interactions obtained from the molecular docking of compound **4**-to TAO without the hydroxide anion. Compound **4** is colored orange and represented as balls and sticks.
